# A Comparison of the Effects of *FATTY ACID DESATURASE 7* and *HYDROPEROXIDE LYASE* on Plant–Aphid Interactions

**DOI:** 10.3390/ijms19041077

**Published:** 2018-04-04

**Authors:** Jiamei Li, Carlos A. Avila, Denise M. Tieman, Harry J. Klee, Fiona L. Goggin

**Affiliations:** 1 Department of Entomology, University of Arkansas, Fayetteville, AR 72701, USA; jxl080@uark.edu; 2 Department of Horticultural Sciences, Texas A&M AgriLife Research, Weslaco, TX 78596, USA; carlos.avila@ag.tamu.edu; 3 Horticultural Sciences Department, University of Florida, Gainesville, FL 32611, USA; dtieman@ufl.edu (D.M.T.); hjklee@ufl.edu (H.J.K.)

**Keywords:** aphid resistance, *Arabidopsis thaliana*, hydroperoxide lyase, *Macrosiphum euphorbiae*, *Myzus persicae*, *Solanum lycopersicum*, ω-3 fatty acid desaturase

## Abstract

The *spr2* mutation in tomato (*Solanum lycopersicum*), which disrupts function of FATTY ACID DESATURASE 7 (FAD7), confers resistance to the potato aphid (*Macrosiphum euphorbiae*) and modifies the plant’s C6 volatile profiles. To investigate whether C6 volatiles play a role in resistance, *HYDROPEROXIDE LYASE* (*HPL*), which encodes a critical enzyme in C6 volatile synthesis, was silenced in wild-type tomato plants and *spr2* mutants. Silencing *HPL* in wild-type tomato increased potato aphid host preference and reproduction on 5-week old plants but had no influence on 3-week old plants. The *spr2* mutation, in contrast, conferred strong aphid resistance at both 3 and 5 weeks, and silencing *HPL* in *spr2* did not compromise this aphid resistance. Moreover, a mutation in the *FAD7* gene in *Arabidopsis thaliana* also conferred resistance to the green peach aphid (*Myzus persicae*) in a genetic background that carries a null mutation in *HPL*. These results indicate that *HPL* contributes to certain forms of aphid resistance in tomato, but that the effects of *FAD7* on aphids in tomato and Arabidopsis are distinct from and independent of *HPL*.

## 1. Introduction

FATTY ACID DESATURASE 7 (FAD7) is an ω-3 fatty acid desaturase (FAD) that is widely similar in sequence throughout the plant kingdom, and that desaturates 16- and 18-carbon fatty acids with two double bonds (C16:2 and C18:2) to generate fatty acids with three double bonds (C16:3 and C18:3) [1,2]. In diverse plant species, expression levels of genes encoding FAD7 and other FADs increase in response to some stresses and decrease in response to others, suggesting that modulation of desaturase activity plays a role in stress adaptation [3,4,5,6]. Moreover, artificial manipulation of desaturase activity through silencing or other genetic modifications alters plants’ susceptibility to a variety of abiotic and biotic stresses, enhancing resistance to some stresses and compromising resistance to others [7,8,9,10]. In short, FADs appear to influence stress resistance.

One form of stress resistance that is negatively correlated with FAD7 activity is resistance to aphids, a group of phloem-feeding insects that include many serious agricultural pests. The *suppressor of prosystemin-mediated responses2* (*spr2*) mutant in tomato, which has a null mutation in *FAD7*, has enhanced resistance to potato aphids (*Macrosiphum euphorbiae*) compared to wild-type plants [10]. Aphid resistance in *spr2* comprises both antixenosis (i.e., decreased host preference) and antibiosis (increased mortality and decreased fecundity). Moreover, population growth of the green peach aphid (*Myzus persicae*) is significantly lower on *Arabidopsis thaliana* mutants with null mutations in *FAD7* than on wild-type controls [10]. These results indicate that the FAD7 enzyme or its products negatively regulate aphid resistance in more than one plant family.

**Figure 1:**
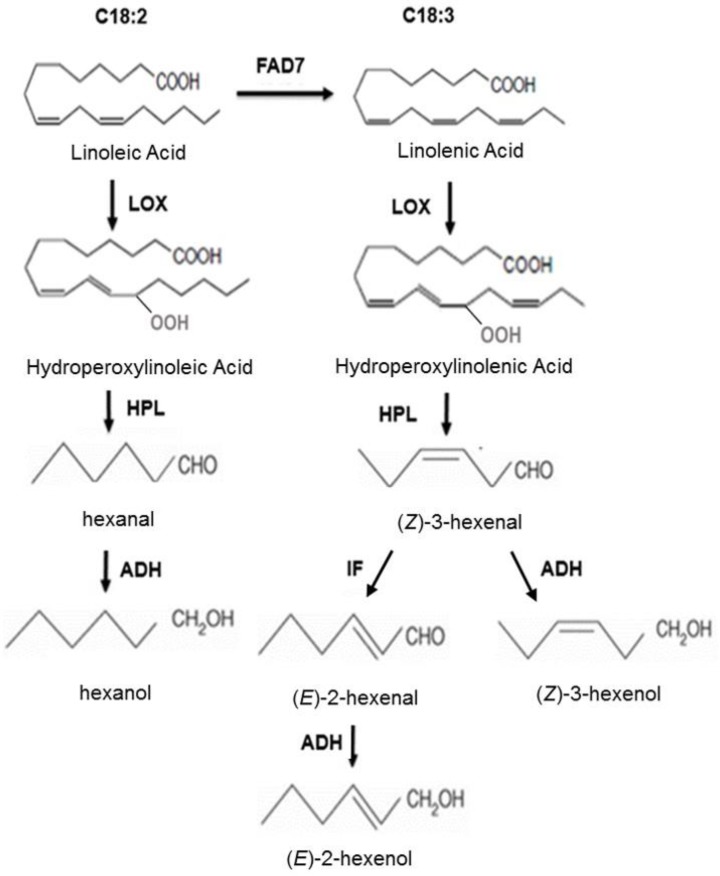
Biochemical pathway for synthesis of C6 volatiles in tomato. In tomato, C6 volatiles are synthesized from the polyunsaturated fatty acids linoleic acid (C18:2) and linolenic acid (C18:3) through the successive action of the enzymes lipoxygenase (LOX), hydroperoxide lyase (HPL), alcohol dehydrogenase (ADH), and isomerization factor (IF). FATTY ACID DESATURASE 7 (FAD7) is an omega-3 FAD that desaturates linoleic acid (C18:2) to generate linolenic acid (C18:3).

One way that FAD7 could possibly influence plant defenses against aphids is by affecting the profile of substrates available to the hydrogen peroxide lyase (HPL) pathway. HPL generates six-carbon aldehydes and alcohols (C6 volatiles) from fatty acid hydroperoxides that are produced from C18:2 and C18:3 by 13-lipoxygenase (13-LOX) (Figure 1) [11]. Loss of function of FAD7 results in decreased C18:3 and increased C18:2, and in tomato, this shift in precursors has been shown to result in dramatically altered C6 volatile composition [12,13]. In particular, decreased levels of C18:3 result in significantly lower (*Z*)-3-hexenal and (*Z*)-3-hexenol than observed in wild-type plants, and enhanced C18:2 levels result in elevated levels of hexanal and hexanol [12,13]. Conversely, overexpression of the *FAD7* gene in tomato increases the production of C6 volatiles derived from 18:3 and decreases the production of 18:2-derived compounds [14]. The impact of altered FAD7 activity on volatile profiles generated by the HPL pathway could potentially alter aphid host selection and/or survival and fecundity on foliage. 

Several lines of evidence indicate that the HPL pathway can influence direct defenses against insects. In in vitro tests, C6 volatiles including hexanal, (*E*)-2-hexenal, hexanol, (*E*)-2-hexenol, and (*Z*)-3-hexenol have been shown to reduce aphid fecundity [15]. Moreover, artificial manipulation of *HPL* gene expression can influence insect resistance. In potato, antisense suppression of a gene encoding a 13-HPL resulted in increased aphid fecundity [16]. Similarly, a null mutation in an *HPL* homolog rendered rice more susceptible to another piercing-sucking insect, the rice brown plant hopper (*Nilaparvata lugens*) [17]. In Arabidopsis, however, overexpression of *HPL* did not influence the host preference, fecundity, or growth of the green peach aphid, even though it resulted in a fifty-fold increase in C6 volatile production [18]. Furthermore, silencing *HPL* in coyote tobacco (*Nicotiana attenuata*) decreased the feeding behavior and growth of a tobacco hornworm (*Manduca sexta*) [19], and overexpression of an *HPL* gene from tea (*Camellia sinensis*) in tomato decreased resistance to another chewing insect, *Prodenia litura* [20]. These studies indicate that the HPL pathway influences insect resistance, but that its effects vary in different species combinations, and that further work is needed to understand the role of HPL in specific plant-insect interactions.

The goals of this study were to determine whether the HPL pathway contributes to direct plant defenses against aphids in wild-type tomato, and whether it is required for the enhanced aphid resistance observed in *spr2* plants, which have impaired FAD7 function. We examined the effects of silencing *HPL* in wild-type plants on aphid infestations at two different stages of plant development (3 and 5 weeks after planting), in comparison with the effects of the *spr2* mutation. Silencing *HPL* increased aphid host preference, offspring production, and offspring survival on 5-week old plants, but did not affect aphid infestations on 3-week old plants, and did not influence the survival of adult aphids at either stage of plant development. In contrast, the *spr2* mutation had a strong suppressive effect on adult survival, fecundity, and offspring survival at both 3 and 5 weeks after planting. Thus, the timing and effects of aphid resistance associated with *HPL* differ from those of *spr2*-dependent resistance. We also developed a tomato line (*spr2HPL-RNAi*) that is deficient in both FAD7 function and *HPL* expression in order to determine if loss of function of HPL would compromise aphid resistance associated with *spr2*. The *spr2HPL-RNAi* line showed similar levels of aphid resistance as the *spr2* parent, despite dramatically reduced levels of *HPL* expression. Similarly, bioassays in Arabidopsis indicated that loss of function of FAD7 could confer aphid resistance even in a genotype that carries a null mutation in *HPL* (the *fad7-1* mutant, which we confirmed to be homozygous for the *hpl* mutation). In summary, our results indicate that both *HPL* and *FAD7* influence antixenosis and antibiosis against aphids in tomato, but that the effects of *FAD7* on aphids are distinct from and independent of *HPL*.

## 2. Results

### 2.1. Confirmation of Silencing of HPL in Tomato

**Figure 2:**
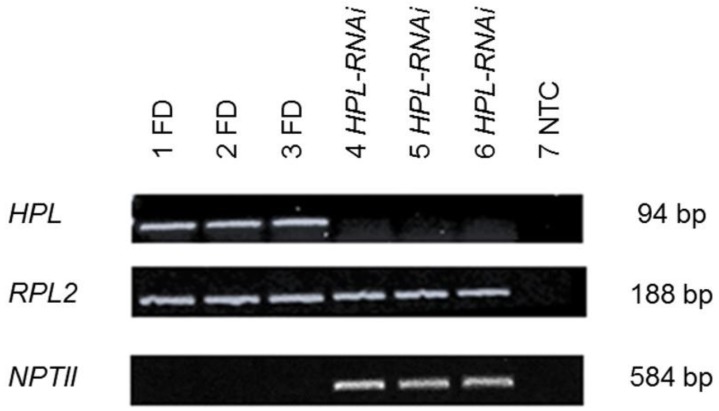
Suppression of *HPL* expression by RNAi. Semiquantitative RT-PCR confirmed reduced *HPL* transcript abundance in *HPL-RNAi* plants (lanes 4–6) compared to untransformed wild-type plants (cv. Flora-Dade, FD, lanes 1–3). The housekeeping gene *Rpl2* was used to confirm uniform RNA quantities across samples. The presence of the transgene in the *HPL-RNAi* line was confirmed by PCR detection of the selective marker *NPTII* in genomic DNA samples from the same plants. NTC = no template control.

A transgenic line in which expression of *HPL* was targeted for silencing by RNA interference (RNAi) [21] was utilized for this study. PCR detection of the kanamycin resistance gene *NPTII* confirmed the presence of the transgene in individuals of the *HPL-RNAi* line, and semi-quantitative PCR confirmed that *HPL* transcript abundance was lower in *HPL-RNAi* plants than in untransformed controls (cv. Flora-Dade) (Figure 2). Analysis of hexanal, (*Z*)-3-hexenal, (*E*)-2-hexenal, and (*Z*)-3-hexen-1-ol levels by gas chromatography (Supplementary Figure S1A–D) also revealed that average C6 volatile production was lower in 5-week-old *HPL-RNAi* plants than in wild-type Flora-Dade.

### 2.2. Influence of HPL and spr2 on Aphid Survival and Fecundity on Tomato

**Figure 3:**
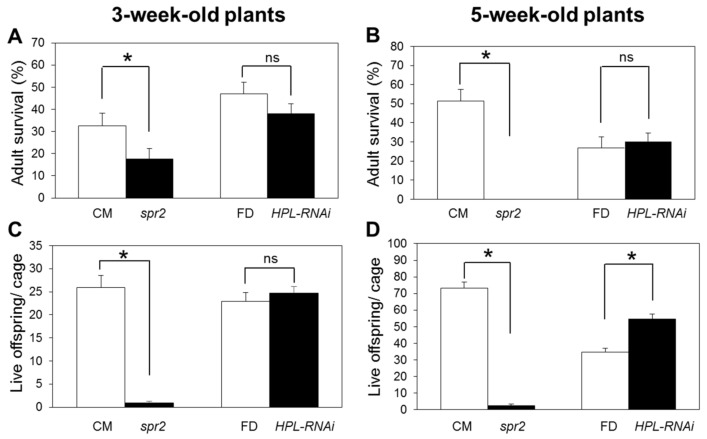
Aphid survival and reproduction on tomato. No-choice assays were performed to assess adult survival (**A**,**B**) and offspring production (**C**,**D**) of caged potato aphids on 3-week (**A**,**C**) and 5-week old plants (**B**,**D**) measured 6 days after inoculation. Asterisks (*) indicate statistically significant differences at α=0.05 according to student’s *t* test, and error bars represent SEM (*n* ≥ 10). Castlemart (CM) and Flora-Dade (FD) are the respective wild-type (WT) controls for *spr2* and *HPL-RNAi*. ns = no significant difference.

Survival of adult aphids and offspring production were measured in no-choice aphid bioassays to assess the effects of silencing *HPL* on aphid antibiosis in a wild-type tomato cultivar (cv. Flora-Dade). For comparison, antibiotic aphid resistance was also quantified in *spr2* compared to its wild-type control (cv. Castlemart). Assays were performed with more than one age of plant (3- and 5-week old plants) just in case the effects of *HPL* on antibiotic defenses varied with plant age. Based on previous reports, HPL activity and volatile production both can vary over the course of development [22,23]. At both stages of development tested here, adult survival was significantly lower on *spr2* than on the wild-type control Castlemart six days after inoculation (3-week old plants: *p* = 0.042; Figure 3A. 5-week old plants: *p* < 0.0001; Figure 3B). The average number of live offspring per cage, which is a measure of adult fecundity, was also more than 50% lower on *spr2* than on Castlemart at either developmental stage (*p* < 0.0001; Figure 3C,D).

In contrast to *spr2*, which influenced aphid infestations at both 3 and 5 weeks, the effects of silencing *HPL* on aphid populations varied with plant age. The live offspring on *HPL-RNAi* were not significantly different from its control Flora-Dade at 3 weeks (*p* = 0.977; Figure 3C), but were approximately 29% higher than on wild-type at five weeks (*p* = 0.005; Figure 3D). Adult survival did not differ between *HPL-RNAi* and Flora-Dade at either age (*p* > 0.05; Figure 3A,B). These data indicate that *HPL* contributes to antibiotic defenses against juvenile aphids in five-week old plants, whereas the *spr2* mutation promotes antibiosis against adults and juveniles at both 3 and 5 weeks after planting.

### 2.3. Influence of HPL and spr2 on Aphid Host Preference on Tomato

**Figure 4:**
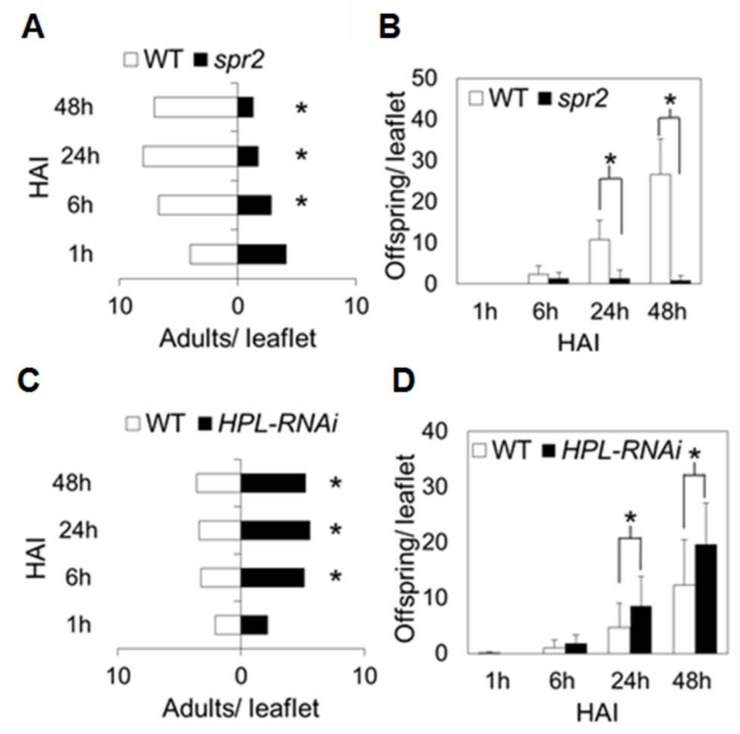
Aphid host preference on tomato. Choice assays were performed to compare aphid settling on *spr2* and *HPL-RNAi* with settling behavior on the respective wild-type (WT) controls: Castlemart and Flora-Dade. Adult potato aphids were offered a choice of two plants from different genotypes (14 aphids per pair of plants; 10 pairs of plants for panels (**A**,**B**), and 15 pairs of plants for panels (**C**,**D**)). Aphid settling behavior was assessed by recording on which plant the adults were located, and how many offspring they produced at 1 h, 6 h, 24 h and 48 h after inoculation (HAI). Asterisks (*) indicate statistically significant differences at α = 0.05 according to Matched pairs one-sided *t*-tests, and error bars represent SEM (*n* ≥ 10).

To measure the effects of *HPL* and *spr2* on aphid settling behavior, pair-wise choice tests were performed to compare the *HPL-RNAi* line or *spr2* to their respective wild-type controls. Choice tests were performed with 5-week-old plants, since both *HPL* and *spr2* influence antibiotic defenses at this life stage (Figure 3). In comparisons between *spr2* and wild-type (cv. Castlemart) plants, aphids initially dispersed onto the two genotypes equally, with no significant difference in adult numbers at 1 h after introduction into the choice arena (*p* = 0.19; Figure 4A). Over time, the numbers of adults on *spr2* decreased and the numbers on wild-type controls increased; compared to wild type plants, *spr2* had significantly fewer aphids at 6 h (*p* = 0.005), 24 h (*p* < 0.0001), and 48 h (*p* < 0.0001) after inoculation (Figure 4A). Offspring were first observed at 6 h, and their abundance was significantly lower on *spr2* compared to wild-type control at 24 h (*p* < 0.0001), and 48 h (*p* < 0.0001) (Figure 4B).

When *HPL-RNAi* was compared to its wild-type control (cv. Flora-Dade), the numbers of adult aphids were significantly higher on *HPL-RNAi* at 6 h (*p* = 0.038), 24 h (*p* = 0.009) and 48 h (*p* = 0.038) (Figure 4C); and the numbers of offspring were significantly higher on *HPL-RNAi* at 24 h (*p* = 0.044) and 48 h (*p* = 0.030) (Figure 4D). These results indicate that aphid host preference is enhanced on the *HPL-RNAi* line compared to wild-type plants, whereas the *spr2* mutation decreases aphid host preference. The effects of *spr2* on aphid host preference appeared to be much greater than the effects of *HPL-RNAi*; for example, at 48 h, the number of juveniles on *spr2* was 97% lower than the number on wild-type plants, whereas silencing *HPL* caused only a 37% change in offspring numbers.

### 2.4. Silencing HPL in the spr2 Mutant

**Figure 5:**
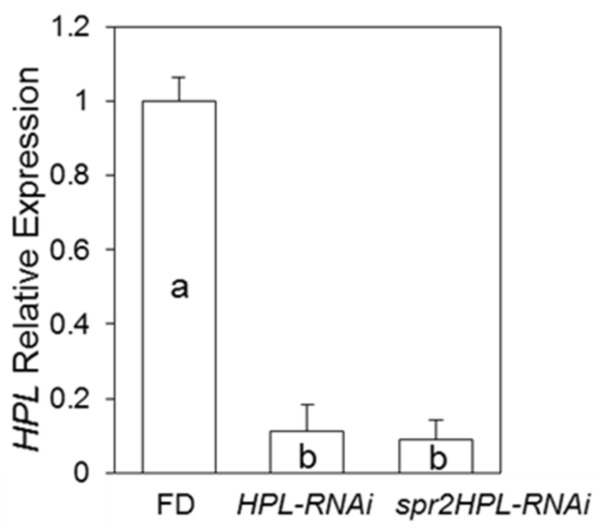
*HPL* Expression in *spr2HPL-RNAi*. RT-qPCR was used to compare *HPL* expression in the wild-type cultivar Flora-Dade (FD), a transgenic line in which the *HPL* gene was silenced (*HPL-RNAi*), and progeny of the *spr2 X HPL-RNAi* cross that were homozygous for the *spr2* mutation in the *FAD7* gene and positive for the *HPL-RNAi* transgene (*spr2HPL-RNAi*). Expression values were normalized using the housekeeping gene *Rpl2* and calculated relative to the wild-type control. Relative expression data were analyzed by one-way ANOVA, and mean separations were performed using Tukey-Kramer HSD. Bars having the same letter are not significantly different at α = 0.05, and error bars represent SEM (*n* ≥ 3).

**Figure 6:**
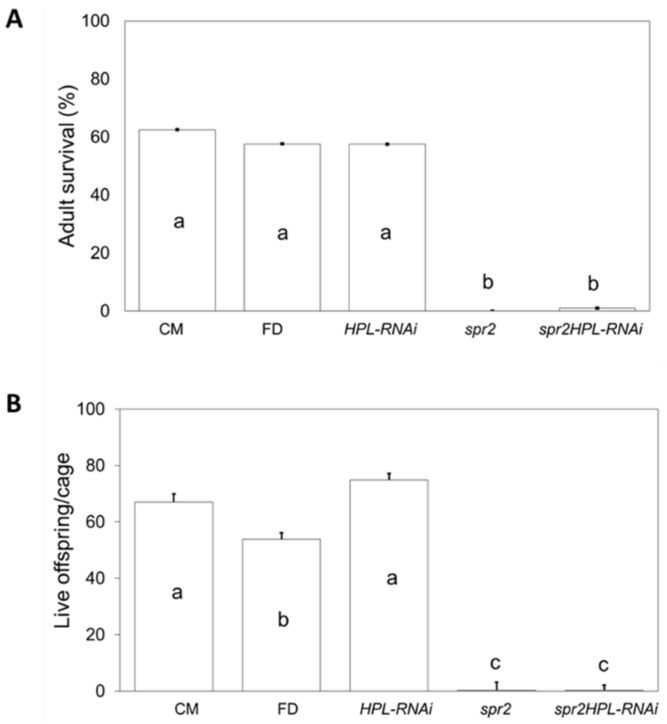
The single and combined effects of impairments in *FAD7* and *HPL* on aphid survival and reproduction. A no-choice assay on 5-week-old plants was used to measure aphid performance on F_2_ progeny of the *spr2 X HPL-RNAi* cross compared to aphid performance on the parental lines (*spr2* and *HPL-RNAi*) and their respective wild-type controls, Castlemart (CM) and Flora-Dade (FD). All progeny used for this assay were confirmed by PCR to be homozygous for the *spr2* mutation and positive for the *HPL-RNAi* transgene. Data was analyzed by one-way ANOVA and Tukey–Kramer HSD. Bars having the same letter are not significantly different at α = 0.05, and error bars represent SEM (*n* ≥ 10).

To determine whether the HPL pathway might contribute to enhanced aphid resistance in *spr2*, the *HPL-RNAi* line was crossed with *spr2*, and PCR genotyping was used to identify F_2_ progeny that were homozygous for the *spr2* mutation and positive for the *HPL-RNAi* construct. RT-qPCR confirmed that *HPL* transcript abundance was significantly reduced in these plants (*spr2HPL-RNAi),* as well as in the *HPL-RNAi* parent line (Figure 5). Compared to the parental lines, the *spr2HPL-RNAi* line had intermediate levels of hexanal, a C6 volatile that accumulates to high levels in *spr2* (Supplementary Figure S1A). No-choice assays were performed to assess aphid performance on *spr2HPL-RNAi* and the parental lines. Bioassays were performed five weeks after planting to focus on a time point when both *spr2* and *HPL-RNAi* impact aphid resistance. As in previous assays, adult survival and live offspring production were significantly lower on *spr2* than on the wild-type control Castlemart (*p* < 0.05; Figure 6A,B), and live offspring production was higher on *HPL-RNAi* than on Flora-Dade (*p* < 0.05; Figure 6B). For both measures of aphid performance, numbers on *spr2HPL-RNAi* were similar to numbers observed on *spr2* (*p* > 0.05, Figure 6A,B), and were significantly lower than numbers observed on the wild-type controls Castlemart and Flora-Dade or on the *HPL-RNAi* parental line (*p* < 0.0001; Figure 6A,B). These data indicate that levels of aphid resistance in *spr2HPL-RNAi* are comparable to levels of resistance in *spr2*, and that silencing *HPL* in an *spr2* background does not compromise aphid resistance mediated by *spr2*.

### 2.5. FAD7 and HPL in Arabidopsis

**Figure 7:**
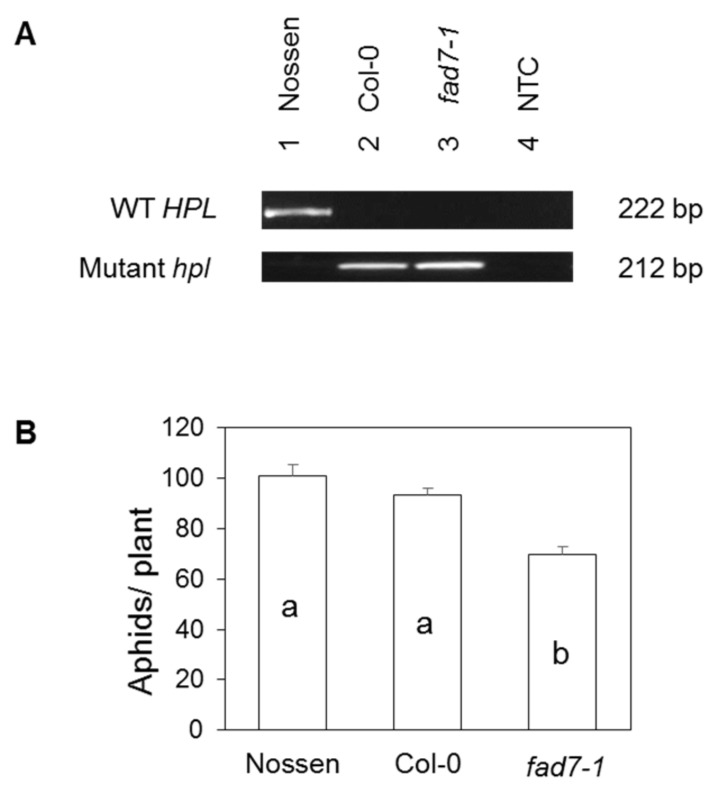
Aphid performance on Arabidopsis genotypes with and without mutations in *FAD7* and *HPL*. PCR was used to determine the presence of wild-type and mutant alleles of the *HPL* gene in Columbia (Col-0), Nossen, and *fad7-1* ((**A**), NTC = no template control). A no-choice test was used to assess performance of the green peach aphid on these genotypes ((**B**), *n =* 20). Aphid numbers were analyzed by one-way ANOVA, and mean separations were performed using Tukey’s HSD. Bars having the same letter are not significantly different at α = 0.05, and error bars represent SEM.

In parallel, we also explored whether the homologous *FAD7* gene in Arabidopsis required a functional copy of the *HPL* gene to influence aphid performance. The Columbia ecotype (Col-0) was previously reported to carry a ten-nucleotide deletion in *HPL* that eliminates the gene’s function [24]. PCR genotyping revealed that the *fad7-1* mutant, which originated from Col-0, is also homozygous for the *hpl* mutation (Figure 7A). This *fad7-1 hpl* mutant supported significantly fewer aphids than the wild-type genotypes Columbia and Nossen (*p* < 0.0001; Figure 7B). Thus, aphid resistance conferred by loss of function of the FAD7 protein is independent of HPL in Arabidopsis as well as in tomato.

Unlike Columbia, the Nossen (Nos) ecotype carries a functional allele of the *HPL* gene (Figure 7A) [24]. Aphid population growth did not differ significantly between these two ecotypes (*p* = 0.2293; Figure 7B). These results suggest that *HPL* does not have a major impact on aphid infestations on Arabidopsis, or that its effects are outweighed by other differences between these two genotypes.

## 3. Discussion

One objective of this study was to determine whether *HPL* contributes to plant defenses against aphids in wild-type tomato plants. Silencing *HPL* expression in wild-type plants (cv. Flora-Dade) had no effect on aphid infestations three weeks after planting (Figure 3), but resulted in enhanced aphid host preference, reproduction, and survival when infestations occurred about five weeks after planting (Figure 3 and Figure 4). These data suggest that *HPL* contributes to both antixenotic and antibiotic defenses against aphids on tomato, and that these defenses vary between 3- and 5-week old plants. Potentially, the activity of the HPL pathway may vary with plant age; for example, in rice, HPL enzyme activity is low in seedlings and peaks twelve weeks after sowing [22]. Therefore, the fact that silencing *HPL* at three weeks had little effect on aphids could be due to relatively low levels of HPL activity in three-week old plants.

A second objective was to determine if the HPL pathway contributes to aphid resistance in the *spr2* mutant, which is defective in a chloroplast-localized fatty acid desaturase FAD7. Like *HPL*, the *spr2* mutation also impacts C6 volatile synthesis because it alters the relative abundance the fatty acid substrates for volatile synthesis. Because the *spr2* mutation enhances C18:2 accumulation and decreases C18:3 synthesis, it promotes production of C18:2-derived volatiles such as hexanal and inhibits accumulation C18:3 derivatives such as (*Z*)-3-hexenol [12,13]. However, the results of our aphid bioassays suggest that products of the HPL pathway do not have a causal role in the enhanced levels of aphid resistance observed in *spr2*. Whereas silencing *HPL* did not influence aphid infestations on three-week old plants, the *spr2* mutation has just as strong an impact on aphids in three-week old seedlings as in five-week old plants (Figure 3). Moreover, when *HPL* is silenced in the *spr2* mutant line, it has no effect on aphid performance on this line (Figure 6). These results indicate that *HPL* expression is not essential to aphid resistance in *spr2*.

Consistent with our observations in tomato, the *fad7-1* mutation in Arabidopsis also enhances aphid resistance even in a genetic background that carries the *hpl* mutation (Figure 7), even though this mutation has previously been shown to suppress C6 volatile synthesis [22]. Thus, FAD7 activity in tomato and Arabidopsis modulates direct defenses against aphids independent of HPL activity. Our results also suggest that *HPL* may not have a strong, direct impact on aphid infestations on Arabidopsis, since aphid population growth was similar on ecotypes with (Col-0) and without (Nossen) the *hpl* mutation (Figure 7). This is consistent with a previous report that overexpression of *HPL* had no influence green peach aphid population increase or host preference on Arabidopsis even though overexpression increased C6 volatile levels by over 40-fold [18].

In conclusion, *HPL* contributes to basal aphid resistance in wild-type tomato plants, but enhanced aphid resistance in mutants with impaired FAD7 function is independent of *HPL* gene expression. In Arabidopsis, although *HPL* aids in indirect defenses against aphids by recruiting parasitoids wasps [18], HPL does not appear to contribute significantly to direct defenses against aphids in the *fad7* mutant. These results indicate that fatty acid metabolism in plants can influence plant-aphid interactions through routes independent of C6 volatile synthesis. It is unlikely that loss of function of FAD7 would impact the nutritional quality of plants for aphids, because polyunsaturated fatty acids are naturally rare in the phloem sap on which aphids feed [25]. Our prior work also indicates that aphid resistance in *spr2* is not influenced by jasmonate signaling, although it requires salicylic acid accumulation and *Non-expressor of Pathogenesis Related Proteins* (*NPR1*) [10]. These findings emphasize the need for further work to understand the mechanisms through which components of primary metabolism including fatty acid desaturation influence plant defense signaling and immunity.

## 4. Materials and Methods

### 4.1. Tomato Culture

Five tomato (*Solanum lycopersicum*) genotypes were used in this study: a mutant line with impaired FAD7 activity called *suppressor of prosystemin-mediated responses2* (*spr2*) [26], a transgenic line silenced for *HPL* (*HPLi-1653-3* [21], referred to here as *HPL-RNAi*), a line deficient in both FAD7 function and *HPL* expression (*spr2HPL-RNAi*), and two wild-type cultivars, Castlemart and Flora-Dade. The *spr2* mutant carries a point mutation that results in loss of function of FAD7 [2], and Castlemart is the genetic background that was originally used to develop *spr2*. The creation of the *HPL-RNAi* line in the tomato cultivar Flora-Dade was previously described [21]. In brief, a 330-bp fragment comprising bases 562–881 of the *HPL* open reading frame in the sense orientation and a 595-bp fragment comprising bases 562–1154 of the *HPL* open reading frame in the antisense orientation were expressed in cv. Flora-Dade under the control of the Figwort mosaic virus 35S in order to induce silencing of *HPL*. The authors previously demonstrated that the *HPL RNAi* line used in this study has significantly reduced *HPL* mRNA accumulation and C6 volatile production in the fruits as well as foliage [21]. Since fruits are not typically produced until at least 8 weeks after planting, this data indicated that silencing was persistent in mature plants. The *spr2HPL-RNAi* line was produced by crossing *spr2* and *HPL-RNAi* (described below). Tomato plants (*Solanum lycopersicum*) were grown in LC1 Sunshine potting mix (Sungro Horticulture, Bellevue, WA, USA) with 15-9-12 Osmocote slow-release fertilizer (Scotts-MiracleGro Company, Marysville, OH, USA) at 23 °C and L16:D8 photoperiod in an environmental growth chamber (Conviron, Winnipeg, MB, Canada), and watered daily with a dilute nutrient solution containing 1000 ppm CaNO_3_ (Hydro Agri North America, Tampa, FL, USA), 500 ppm MgSO_4_ (Giles Chemical Corp, Waynesville, NC, USA), and 500 ppm 4-18-38 Gromore fertilizer (Gromore, Gardena, CA, USA).

### 4.2. Development of the spr2HPL-RNAi Line

A tomato line with impairments in both *FAD7* and *HPL* was developed by manually transferring pollen from *HPL-RNAi* to *spr2* and screening the (*spr2* × *HPL-RNAi*) F_2_ generation for individuals that were positive for the *HPL-RNAi* transgene and homozygous for the *spr2* mutation. Screening for the *spr2* mutation was performed by PCR using two allele-specific primer sets that target a single nucleotide polymorphism as described by Avila et al. [10]. Presence of the *HPL*-*RNAi* transgene in F_2_ plants was determined by amplifying the selectable marker *NPTII* (*Neomycin phosphotransferase II*) using forward (5′-GCAATATCACGGGTAGCCAA-3′) and reverse (5′-GCCGTGTTCCGGCTGTCA-3′) primers. *NPTII* PCR was performed at 95 °C for 5 min; 95 °C for 45 s, 50 °C for 45 s, and 72 °C for 45 s (30 cycles); and final extension at 72 °C for 5 min.

### 4.3. Arabidopsis Culture and Materials Development

Arabidopsis plants (*Arabidopsis thaliana*) were grown in a peat, vermiculite, perlite (4:3:2 ratio, Sungro Horticulture, Bellevue, WA, USA) soil mixture supplemented with 15-9-12 Osmocote Plus fertilizer (Scotts-MiracleGro Company, Marysville, OH, USA) at 23 °C and L13:D11 photoperiod in a growth chamber (Conviron, Winnipeg, MB, Canada). The plants were fertilized weekly with Miracle Gro^®^ all-purpose plant food (Scotts-MiracleGro Company, Marysville, OH, USA). Arabidopsis ecotype Columbia (Col-0, CS70000) was obtained from the Arabidopsis Biological Resource Center (Ohio State University, Columbus, OH, USA), and the Nossen ecotype and the *fad7-1gl1* mutant (developed in a Col-0 genetic background) were obtained from Dr. Jyoti Shah (University of North Texas). Because the *fad7-1gl1* mutant carries a mutation (*gl1*) in a gene required for trichome production (*GLABRA1*) in addition to a mutation in *FAD7*, this mutant was crossed with Col-0 to develop another line (*fad7-1*) with impaired FAD7 function but normal trichome development. In the F_2_ generation, plants with trichomes were screened by PCR with primer sets specific to the mutant and wild-type alleles of *FAD7* to select for plants homozygous for the mutant *fad7-1* allele [27]. Plants that lacked the *gl1* mutant phenotype and that were homozygous for the *fad7-1* mutation were then propagated to generate seeds for subsequent assays. All plants were observed to confirm the presence of trichomes before they were used for experiments.

### 4.4. Identification of the HPL Mutation in the Arabidopsis fad7-1 Mutant

The *fad7-1* mutant in Arabidopsis was screened by PCR for the presence of a 10-bp deletion (from 161 bp to 170 bp) in the *HYDROPEROXIDE LYASE* (*HPL*) gene that occurs naturally in the ecotype Columbia (Col-0), and that results in a non-functional HPL protein [24]. Col-0 was included as a positive control for the mutant allele, and the Nossen ecotype was included as a positive control for the wild-type *HPL* allele. Genomic DNA was extracted using an extraction buffer that was made by diluting Edwards solution (200 mM Tris-HCl (pH 7.5), 250 mM NaCl, 25 mM EDTA, and 0.5% SDS) by 10-fold with TE buffer (10 mM Tris-HCl (pH 8) and 1 mM EDTA) [28,29]. Each sample was used for two separate PCR reactions: one with a primer set that amplifies only the wild-type *HPL* allele (At4g15440.1) (forward 5′-GGACCGTTTAGATTACTTCTGGTT-3′, reverse 5′-CGGAAGTCTCCGATGAGAAC-3′), and another reaction with a primer set that specifically targets the mutant *hpl* allele with the 10 nts deletion (5′-GACCGTTTAGATTCCAAGGAC-3′, reverse 5′-CGGAAGTCTCCGATGAGAAC-3′). PCR amplification was performed at 95 °C for 5 min, followed by 30 cycles of 95 °C for 45 s; 55 °C for 45 s, and 72 °C for 45 s, and a final extension at 72 °C for 5 min. PCR products were separated by electrophoresis on 1% agarose gels.

### 4.5. RNA Isolation and Gene Expression Analysis

For analysis of gene expression, total RNA was extracted from approximately 100 mg of flash-frozen leaf tissue using TRIzol reagent and chloroform (Invitrogen Corp., Carlsbad, CA, USA) using the manufacturer’s instructions. cDNA was synthesized from 1 μg of total RNA per sample using Superscript III reverse transcriptase and oligo dT_(18)_ primers in a 20 µL reaction volume (Invitrogen Corp., Carlsbad, CA, USA). Transcript abundance in the cDNA was then quantified by semi-quantitative PCR or real-time PCR. For semi-quantitative PCR, 50 ng of cDNA was used as a template and the final concentration for each primer was 0.4 µM. The PCR program was: 95 °C for 5 min; 95 °C for 30 s, 52 °C for 30 s, and 72 °C for 30 s (22 cycles); and final extension at 72 °C for 5 min. PCR products were detected on a 1% agarose gel. Real-time quantitative PCR was performed on 2 µL of cDNA in a 20 µL reaction volume using the QuantiTect SYBR Green PCR Kit (Qiagen, Inc., Valencia, CA, USA) on a StepOnePlus Real-time PCR system (Applied Biosystems, Foster City, CA, USA). The RT-qPCR program was: 95 °C activation for 10 min, followed by 40 cycles of amplification and quantification (denaturation at 95 °C for 15 s, annealing at 52 °C for 30 s, extension 72 °C for 30 s with a single fluorescence detection). Melting curves were generated at 60–95 °C with a heating rate of 0.3 °C per second. Three biological replicates for each genotype and two technical replicates for each biological replicate were used. Transcript abundance of tomato *HPL* (Solyc07g049690; GenBank accession AF230372.1) was measured using primers previously described by Shen and coworkers [21] (5′-AGCTACGGATTGCCGTTAGT-3‘/5′-TTTCCATTCTCTTGGTGAAGAA-3′). Data were normalized to the expression levels of the endogenous control *Ribosomal Protein L2 (RPL2)* using primers previously described by Avila and coworkers [10] (5′-GAGGGCGTACTGAGAAACCA-3′/5′-CTTTTGTCCAGGAGGTGCAT-3′). Gene expression was calculated relative to the wild-type control for each genotype comparison using the methodology described by Pfaffl [30].

### 4.6. Aphid Bioassays

#### 4.6.1. Insect Materials

Potato aphids (*Macrosiphum euphorbiae*) were reared on an aphid-susceptible tomato cultivar (cv. BHN876, potato (*Solanum tuberosum* Linnaeus), and Jimson weed (*Datura stramonium* Linnaeus) plants at 20 °C and 16-h light photoperiod. Green peach aphids (*Myzus persicae*) were reared on an aphid-susceptible cabbage cultivar (*Brassica oleracea* var. Joychoi) at ~23 °C and 16-h light photoperiod.

#### 4.6.2. Aphid Survival and Fecundity on Tomato

No-choice assays were performed to evaluate potato aphid survival and fecundity. Wingless adult potato aphids within 24 h of emergence to adulthood were confined to single leaflets of intact plants using clip cages (4 adults per cage, 2 cages per plant, 10–15 replicate plants per genotype), and the numbers of living and dead adults and offspring were recorded at six days after infestation (6-DAI). Both 3- and 5-week-old plants were inoculated to determine if aphid resistance varied with age. The positions of the cages were standardized for all plants in each assay; cages were placed on the terminal leaflet of the 2–3 fully-expanded leaves below the apical meristem. Plants were maintained in a growth chamber at 23 °C and 16L: 8D photoperiod during the bioassay.

#### 4.6.3. Aphid Host Preference on Tomato

Settling behavior of the potato aphid was measured on intact tomato plants by placing adult aphids on choice arenas that allowed them to move back and forth between paired tomato genotypes. Each arena consisted of a Styrofoam platform (15 cm diameter) that was placed underneath two paired leaflets: the terminal leaflet of the third fully expanded leaf below the apical meristem of each of the paired plants. Wingless adult potato aphids within 24 h of emergence to adulthood (14 adults per arena) were placed between the leaflets and confined to the arena using a vented petri dish lid with a soft gasket that prevented damaging the petioles (Supplementary Figure S2). The number of adult aphids on each plant were recorded at 1 h, 6 h, 24 h and 48 h after release. Offspring production was also monitored because it is a well-established marker of host plant acceptance [31]. In each experiment, ten to fifteen replicate pairs per combination of genotypes were tested using five-week-old tomato plants, and each experiment was performed at least twice.

#### 4.6.4. Aphid Survival and Fecundity on Arabidopsis

To measure aphid performance on Arabidopsis, plants were inoculated with the green peach aphid (3 wingless newly-emerged adults/plant; 15 plants/genotype) when first flower buds were visible (developmental stage 5.1 according to [32]). After infestation, plants were covered with sleeve cages and maintained for 7 days in a growth chamber (23 °C; 65% relative humidity; L13:D11 photoperiod). The numbers of live and dead adults and offspring aphids on each plant were scored 7 days after infestation (DAI) in this no-choice assay.

### 4.7. Statistical Analysis

All statistical analyses were done with JMP^®^ v.11 (SAS Institute Inc., Cary, NC, USA). Host preference assays were analyzed by matched pairs one sided *t*-tests within each time point, and no-choice assays were analyzed by one-way ANOVA, with α = 0.05.

## Supplementary Materials

The following are available online at http://www.mdpi.com/1422-0067/19/4/1077/s1. Figure S1: Comparison of C6 volatile levels in five-week-old plants with modifications in fatty acid desaturation and/or HYDROPEROXIDE LYASE expression. Figure S2: Design of choice assays to study aphid host preference on tomato.

## References

[1] Browse J., McCourt P., Somerville C. (1986). A mutant of Arabidopsis deficient in C_18:3_ and C_16:3_ leaf lipids. Plant Physiol..

[2] Li C., Liu G., Xu C., Lee G.I., Bauer P., Ling H.Q., Ganal M.W., Howe G.A. (2003). The tomato *suppressor of prosystemin-mediated responses2* gene encodes a fatty acid desaturase required for the biosynthesis of jasmonic acid and the production of a systemic wound signal for defense gene expression. Plant Cell.

[3] Gibson S., Arondel V., Iba K., Somerville C. (1994). Cloning of a temperature-regulated gene encoding a chloroplast omega-3 desaturase from *Arabidopsis thaliana*. Plant Physiol..

[4] Berberich T., Harada M., Sugawara K., Kodama H., Iba K., Kusano T. (1998). Two maize genes encoding omega-3 fatty acid desaturase and their differential expression to temperature. Plant Mol. Biol..

[5] Kodama H., Nishiuchi T., Seo S., Ohashi Y., Iba K. (2000). Possible involvement of protein phosphorylation in the wound-responsive expression of *Arabidopsis* plastid ω-3 fatty acid desaturase gene. Plant Sci..

[6] Dong C.-J., Cao N., Zhang Z.-G., Shang Q.-M. (2016). Characterization of the fatty acid desaturase genes in cucumber: Structure, phylogeny, and expression patterns. PLoS ONE.

[7] Murakami Y., Tsuyama M., Kobayashi Y., Kodama H., Iba K. (2000). Trienoic fatty acids and plant tolerance of high temperature. Science.

[8] Im Y.J., Kim M.S., Yang K.Y., Kim Y.H., Back K., Cho B.H. (2004). Antisense expression of a ω-3 fatty acid desaturase gene in tobacco plants enhances susceptibility against pathogens. Can. J. Bot..

[9] Yara A., Yaeno T., Hasegawa M., Seto H., Montillet J.-L., Kusumi K., Seo S., Iba K. (2007). Disease resistance against *Magnaporthe grisea* is enhanced in transgenic rice with suppression of ω-3 fatty acid desaturases. Plant Cell Physiol..

[10] Avila C.A., Arevalo-Soliz L.M., Jia L., Navarre D.A., Chen Z., Howe G.A., Meng Q.W., Smith J.E., Goggin F.L. (2012). Loss of function of FATTY ACID DESATURASE 7 in tomato enhances basal aphid resistance in a salicylate-dependent manner. Plant Physiol..

[11] Matsui K. (2006). Green leaf volatiles: Hydroperoxide lyase pathway of oxylipin metabolism. Curr. Opin. Plant Biol..

[12] Canoles M.A., Beaudry R.M., Li C., Howe G. (2006). Deficiency of linolenic acid in *Lefad7* mutant tomato changes the volatile profile and sensory perception of disrupted leaf and fruit tissue. J. Am. Soc. Hortic. Sci..

[13] Sanchez-Hernandez C., Lopez M.G., Delano-Frier J.P. (2006). Reduced levels of volatile emissions in jasmonate-deficient *spr2* tomato mutants favour oviposition by insect herbivores. Plant Cell Environ..

[14] Domínguez T., Hernández M.L., Pennycooke J.C., Jiménez P., Martínez-Rivas J.M., Sanz C., Stockinger E.J., Sánchez-Serrano J.J., Sanmartín M. (2010). Increasing ω-3 desaturase expression in tomato results in altered aroma profile and enhanced resistance to cold stress. Plant Physiol..

[15] Hildebrand D.F., Brown G.C., Jackson D.M., Hamilton-Kemp T.R. (1993). Effects of some leaf-emitted volatile compounds on aphid population increase. J. Chem. Ecol..

[16] Vancanneyt G., Sanz C., Farmaki T., Paneque M., Ortego F., Castanera P., Sanchez-Serrano J.J. (2001). Hydroperoxide lyase depletion in transgenic potato plants leads to an increase in aphid performance. Proc. Natl. Acad. Sci. USA.

[17] Tong X., Qi J., Zhu X., Mao B., Zeng L., Wang B., Li Q., Zhou G., Xu X., Lou Y. (2012). The rice hydroperoxide lyase OsHPL3 functions in defense responses by modulating the oxylipin pathway. Plant J..

[18] Chehab E.W., Kaspi R., Savchenko T., Rowe H., Negre-Zakharov F., Kliebenstein D., Dehesh K. (2008). Distinct roles of jasmonates and aldehydes in plant-defense responses. PLoS ONE.

[19] Halitschke R., Ziegler J., Keinanen M., Baldwin I.T. (2004). Silencing of hydroperoxide lyase and allene oxide synthase reveals substrate and defense signaling crosstalk in *Nicotiana attenuata*. Plant J..

[20] Xin Z., Zhang L., Zhang Z., Chen Z., Sun X. (2014). A tea hydroperoxide lyase gene, *CsiHPL1*, regulates tomato defense response against *Prodenia litura* (Fabricius) and *Alternaria alternate* f. sp. *Lycopersici* by modulating green leaf volatiles (GLVs) release and jasmonic acid (JA) gene expression. Plant Mol. Biol. Report..

[21] Shen J., Tieman D., Jones B., Taylor G., Schmelz E., Huffaker A., Bies D., Chen K., Klee H.J. (2014). A 13-lipoxygenase, TomloxC, is essential for synthesis of C5 flavour volatiles in tomato. J. Exp. Bot..

[22] Liu X., Li F., Tang J., Wang W., Zhang F., Wang G., Chu J., Yan C., Wang T., Chu C. (2012). Activation of the jasmonic acid pathway by depletion of the hydroperoxide lyase OsHPL3 reveals crosstalk between the HPL and AOS branches of the oxylipin pathway in rice. PLoS ONE.

[23] Hanley M.E., Girling R.D., Felix A.-E., Olliff E.D., Newland P.L., Poppy G.M. (2013). Olfactory selection of *Plantago lanceolata* declines with seedling age. Ann. Bot..

[24] Duan H., Huang M.-Y., Palacio K., Schuler M.A. (2005). Variations in CYP74B2 (hyperoxide lyase) gene expression differentially affect hexenal signaling in the Columbia and Landsberg erecta ecotypes of *Arabidopsis*. Plant Physiol..

[25] Madey E., Nowack L., Thompson J. (2002). Isolation and characterization of lipid in phloem sap of canola. Planta.

[26] Howe G.A., Ryan C.A. (1999). Suppressors of systemin signaling identify genes in the tomato wound response pathway. Genetics.

[27] Vaughn K.L., Avila C.A., Padilla-Marcia C.S., Goggin F.L. (2014). Development of *fad7-1* single mutant *Arabidopsis thaliana* plants that are resistant to aphids. Discovery.

[28] Edwards K., Johnstone C., Thompson C. (1991). A simple and rapid method for the preparation of plant genomic DNA for PCR analysis. Nucleic Acids Res..

[29] Kasajima I., Ide Y., Ohkama-Ohtsu N., Hayashi H., Yoneyama T., Fujiwara T. (2004). A protocol for rapid DNA extraction from *Arabidopsis thaliana* for PCR analysis. Plant Mol. Biol. Report..

[30] Pfaffl M.W. (2001). A new mathematical model for relative quantification in real-time RT-PCR. Nucleic Acids Res..

[31] Powell G., Tosh C.R., Hardie J. (2006). Host plant selection by aphids: Behavioral, evolutionary, and applied perspectives. Annu. Rev. Entomol..

[32] Boyes D.C., Zayed A.M., Ascenzi R., McCaskill A.J., Hoffman N.E., Davis K.R., Gӧrlach J. (2001). Growth stage-based phenotypic analysis of Arabidopsis: A model for high throughput functional genomics in plants. Plant Cell.

