# Supplementary material for: A Comparison of the Effects of FATTY ACID DESATURASE 7 and HYDROPEROXIDE LYASE on Plant–Aphid Interactions
Source: Int J Mol Sci. 2018 Apr 4;19(4):1077. doi: 10.3390/ijms19041077 (PMC5979546; doi:10.3390/ijms19041077)
Supplement: Supplementary file 1 [file ijms-19-01077-s001.pdf]

Supplementary figure S12. Comparison of C6 volatile levels in five-week-old plants with impairments in fatty acid desaturation and/or hydroperoxide lyase expression. The C6 volatile levels ((Hexanal (A), (*Z*)-3-hexenal (B), (*E*)-2-hexenal (C), and (*Z*)-3-hexen-1-ol (D)) were compared in 5-week old plants with a mutation in *Fatty Acid Desaturase 7* (*spr2*), silencing of hydroperoxide lyase expression (*HPL-RNAi*), or impairments in both fatty acid desaturation and *HPL* expression (*spr2HPL-RNAi*). The wild-type cultivars Castlemart (CM) and Flora-Dade (FD) were included as controls for *spr2* and *HPL-RNAi* respectively. The terminal leaflet of the third fully expanded leaf below the apical meristem was collected, weighed, and flash-frozen in liquid nitrogen (n≥3 per genotype), and volatiles were collected and analyzed as previously described [1]. In brief, samples were spiked with the internal standard pentadecane-d_{32} and ground in 50% $CaCl_2$ solution. Headspace volatiles were collected from crushed foliage by a solid-phase microextraction and analyzed by gas chromatography. Volatiles were quantified based on the internal standard and identified by comparing retention times and mass spectra to known standards (SigmaAldrich, St. Louis, MO). Data were analyzed by one-way ANOVA, and mean separations were performed using Tukey-Kramer HSD. Asterisks represent differences that are statistically significant at α=0.05.

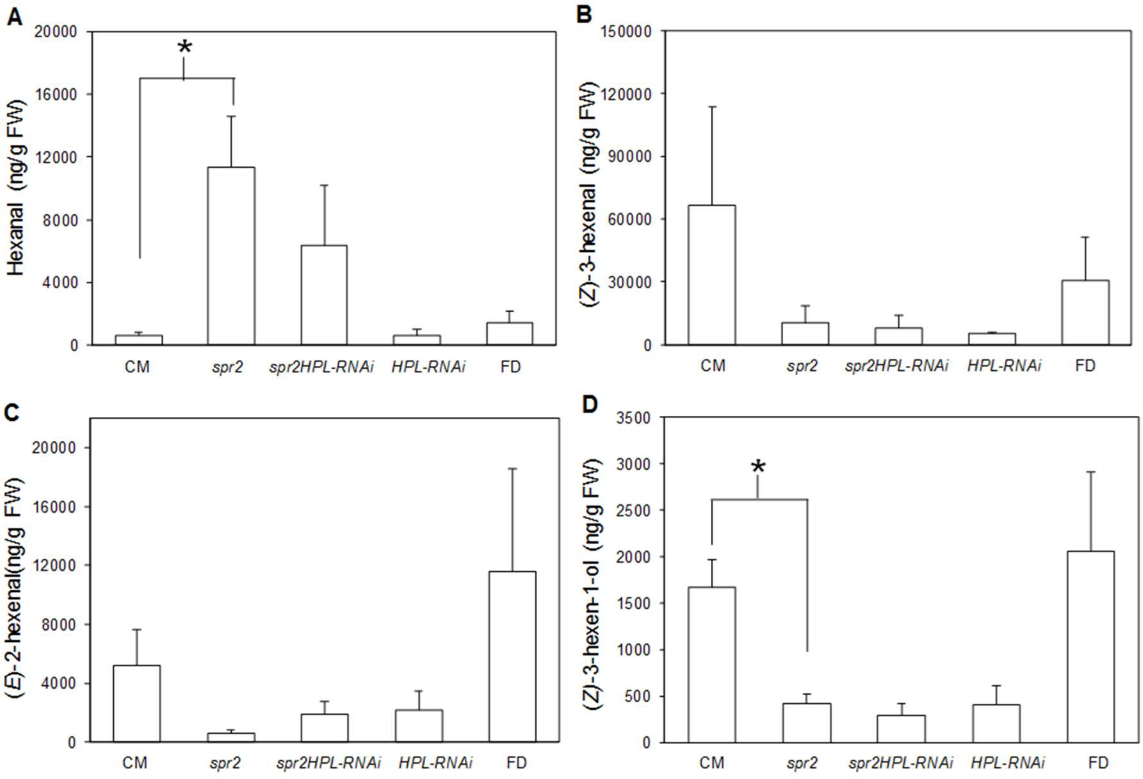

Supplementary figure S2. Design of choice assays to study aphid host preference on tomato. Aphids were offered a choice arena to move back and forth between two plants from different genotypes. Wingless adult potato aphids within 24 h of emergence to adulthood (14 adults per arena) were placed between the leaflets and confined to the arena. Aphid settling behavior was assessed by recording on which plant the adults were located, and how many offspring they produced after inoculation.

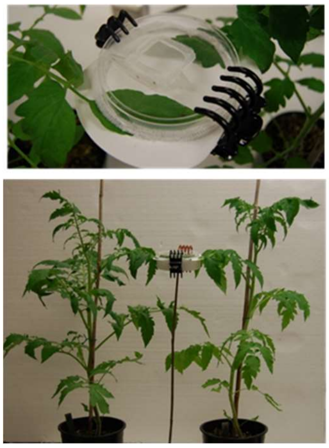

1. Tieman, D.M; Zeigler, M; Schmelz, E.A.; Taylor, M.G.; Bliss, P.; Kirst, M.; Klee, H.J. Identification of loci affecting flavour volatile emissions in tomato fruits. *J. Exp. Bot.* **2006**, *57*, 887–896.
